# Dietitians’ Adherence and Perspectives on the European Association for the Study of Obesity (EASO) and the European Federation of the Associations of Dietitians (EFAD) Recommendations for Overweight and Obesity Management: A Mixed-Methods Study

**DOI:** 10.3390/nu17172736

**Published:** 2025-08-23

**Authors:** Odysseas Androutsos, Hilda Mulrooney, Vaios Svolos, Antonis Vlassopoulos, Elisabeth Govers, Maria Hasssapidou

**Affiliations:** 1 Laboratory of Clinical Nutrition and Dietetics, Department of Nutrition and Dietetics, School of Physical Education, Sports Science and Dietetics, University of Thessaly, 42132 Trikala, Greece; vaiossvolos@gmail.com; 2 ESDN-Obesity, European Federation of the Associations of Dietitians (EFAD), The Netherlandsantonisvlass@gmail.com (A.V.); e.govers112@upcmail.nl (E.G.); mnhass@gmail.com (M.H.); 3 School of Human Sciences, London Metropolitan University, London N7 8DB, UK; 4 School of Life Sciences, Pharmacy & Chemistry, Kingston University, Kingston-upon-Thames KT1 2EE, UK; 5 School of Medicine, Dentistry and Nursing, College of Medical, Veterinary and Life Sciences, University of Glasgow, Glasgow G12 8QF, UK; 6 Department of Food Science & Human Nutrition, Agricultural University of Athens, 11855 Athens, Greece; 7 Knowledge Centre of Obesity Dietitians, Amsterdam, The Netherlands; 8 Department of Nutritional Sciences and Dietetics, International Hellenic University, 57400 Thessaloniki, Greece

**Keywords:** obesity, medical nutrition therapy, clinical practice, guideline dissemination, obesity management, dietetic practice, dietitian, nutritionist, knowledge

## Abstract

Introduction: Recent guidelines developed by the European Association for the Study of Obesity (EASO) and the European Federation of the Associations of Dietitians (EFAD) focused on the dietetic management of obesity in adults. The present study aimed to explore the perspectives of healthcare professionals regarding these guidelines. Methods: In total, 85 registered dietitians/nutritionists from Greece, the Netherlands, the Republic of Ireland, and the United Kingdom completed an online survey, and 10 were interviewed, in February–March 2023. Demographic data were also collected. Results: Awareness of the EASO-EFAD guidelines among registered dietitians/nutritionists was moderate (57.6%), but only 20% had read them in full. Dietitians with higher education and relevant experience were more likely to have read the guidelines. Less than half reported that key evidence-based recommendations, such as individualized medical nutrition therapy and intensive behavioral interventions, are already included in national guidance. Recommendations like portfolio or DASH diets, partial meal replacements, and calorie restriction were less commonly part of national guidance/usual practice. A small percentage of participants described their adoption of several nutritional approaches novel to them. These included the portfolio dietary pattern, partial meal replacements, and intermittent fasting or continuous calorie restriction. For some Irish dietitians, prioritizing weight as the main outcome conflicted with their emphasis on overall health and individualized nutrition therapy. Other barriers of recommendation implementation included exclusive availability in English, rapid changes in obesity management, staffing shortages, limited multidisciplinary collaboration, and inconsistent knowledge among healthcare providers. Conclusions: The present study identified gaps in the adoption of the EASO-EFAD guidelines into dietetic/clinical practice. EFAD will develop strategies to disseminate these guidelines at different levels of stakeholders (national/local authorities, dietitians/nutritionists, and patients).

## 1. Introduction

The escalating prevalence of obesity across Europe has necessitated the development of comprehensive and unified recommendations for its management [1]. Obesity, a multifactorial chronic disease, is intricately linked to a range of comorbidities, including cardiovascular disease, type 2 diabetes, and certain types of cancer [2]. European healthcare systems are under pressure to implement effective strategies for its prevention and management, with a pivotal role assigned to healthcare professionals (HCPs) in translating policy into practice. Considering the gaps that were previously identified at a European level, recent European guidelines on medical nutritional therapy (MNT) for adults living with obesity emphasize a person-centered approach, advocating for personalized interventions, dietary patterns, and non-dieting strategies that prioritize well-being over calorie restriction or rigid dietary plans, an approach advocated as effective for fostering better quality of life and promoting healthier body image perceptions [3,4].

Overweight and obesity represent among the most pressing public health challenges in Europe, with prevalence rates continuing to rise despite decades of prevention strategies. Current estimates suggest that more than half of adults in many European Union member states live with overweight or obesity, placing substantial burdens on healthcare systems, social structures, and economies. Importantly, obesity is associated not only with metabolic and cardiovascular diseases but also with mental health disorders, musculoskeletal conditions, and reduced quality of life, underscoring the complexity of its management [5,6].

While evidence-based guidelines for overweight and obesity management exist at both international and national levels [7,8], research has consistently shown that these are not fully implemented in clinical practice. Gaps in guideline adherence are often attributed to limited awareness, insufficient training, and the absence of structured systems to support multidisciplinary collaboration. For example, lifestyle modification programs, despite being the cornerstone of obesity treatment, are underutilized in many countries due to limited institutional support, low reimbursement rates, and lack of integration into routine care pathways. This mismatch between available evidence and real-world practice highlights the importance of examining how dietitians and other professionals perceive and apply guideline recommendations in their daily work [3,9,10,11].

The European Association for the Study of Obesity (EASO) and the European Federation of the Associations of Dietitians (EFAD) guidelines provide one of the most comprehensive frameworks for medical nutrition therapy in adult overweight and obesity management [4]. They not only summarize the best available evidence but also encourage innovative dietary strategies, such as partial meal replacements, intermittent fasting, and dietary patterns including the DASH and portfolio diets. Nevertheless, the integration of such recommendations into national guidelines and everyday practice is far from universal [4]. Previous European surveys have highlighted considerable heterogeneity in the structure, content, and accessibility of obesity-related guidelines across countries. In some regions, guidelines remain outdated, fragmented, or available only in English, creating practical barriers to adoption [3,12]. Moreover, a shortage of trained dietitians and limited opportunities for multidisciplinary teamwork further constrain implementation [13]. In this context, understanding dietitians’ experiences is essential to ensure that guideline recommendations are both feasible and acceptable in real-world practice.

The perspectives of dietitians, nutritionists, and other HCPs are critical in understanding the feasibility, acceptance, and barriers to implementing these recommendations. HCPs’ experiences offer insights into the operationalization of guidelines, from navigating complex patient needs to addressing systemic limitations such as time constraints and inadequate training [14]. Engaging with HCPs provides a dynamic platform to explore these perspectives, fostering the identification of gaps and potential adaptations needed for successful implementation.

The objective of the present study was to investigate the perspectives of dietitians and nutritionists on the new European guidelines by the European Association for the Study of Obesity (EASO) and the European Federation of the Associations of Dietitians (EFAD) for overweight and obesity management [4].

## 2. Materials and Methods

### 2.1. Ethical Clearance

The present study was approved by the Bioethics Committee of the Department of Nutrition-Dietetics of the University of Thessaly, Greece (4/08.07.2021). All participants in the study participated after giving informed consent. All procedures followed were in line with the Declaration of Helsinki and the GDPR rules.

### 2.2. Study Design, Participants, and Tools

The present study was based on an online survey and optional semi-structured interviews with dietitians and nutritionists from Greece, the Netherlands, the Republic of Ireland, and the United Kingdom in February–March 2023. A total of 85 dietitians/nutritionists completed the online questionnaires, and 10 were interviewed. Inclusion criteria for dietitians and nutritionists in the study were based on their (i) official registration as nutritionists/dietitians within their country and (ii) active participation in obesity management of adults. Recruitment was conducted through professional networks (e.g., EFAD) and snowball sampling.

Dietitians/nutritionists self-reported their awareness and perspectives on the European recommendations for obesity management in adults, developed by EASO-EFAD [4], using a bespoke questionnaire that was developed for the needs of this study. A draft questionnaire was compiled by senior academic and dietetic staff with expertise in obesity management. The content validity of the survey questionnaire was then assessed by members of the University of Thessaly and Kingston University teams (O.A. and H.M., respectively). The survey collected information on participant demographic characteristics, the knowledge of dietitians/nutritionists about the European recommendations, their thoughts on each recommendation statement, and the perceived barriers and facilitators for the implementation of these recommendations in clinical practice. The study questionnaire can be found in Appendix A. Those interested in doing so could also take part in an optional semi-structured interview, held online or by telephone and audio-recorded. An interview topic guide was used for consistency in all interviews (see Appendix B), and field notes were taken. Interviews sought to identify the main changes to practice that would be required to adopt the recommendations and barriers to such changes.

As this was an exploratory mixed-methods study, no formal power calculation was conducted. The sample size was determined pragmatically to capture preliminary cross-country perspectives. The qualitative component, comprising 10 interviews, is consistent with methodological guidance indicating that thematic saturation is often achieved within 6–12 interviews in exploratory studies.

### 2.3. Statistical Analysis

Quantitative data are presented as absolute (n) and relative (%) frequencies. Comparisons between groups were conducted using chi-square tests. For all statistical analyses the SPSS software was used (SPSS V26 software package, IBM, Armonk, NY, USA), and *p* < 0.05 was set as the level of significance.

### 2.4. Thematic Analysis

Audio recordings of interviews were transcribed verbatim, and thematic analysis was carried out to identify key themes, taking a deductive approach to identify barriers in the implementation of EASO/EFAD recommendations in clinical practice [15]. The transcripts and interview notes were read over multiple times to manually identify codes and themes through an iterative process.

## 3. Results

### 3.1. Questionnaire Survey

In total, 85 dietitians and nutritionists (74.1% females) completed the online survey, and 10 were interviewed. The majority (34.1%) were aged 40–49 years, while 29.4% were 30–39 years, 22.4% were <30 years, and the remainder were ≥50 years. Over half (52.9%) had completed postgraduate studies. Working experience of weight management was high; 36.5% had 0–5 years of experience, 22.4% had 5–10 years, 17.6% had 10–15 years, and 15.3% had >20 years.

**Figure 1:**
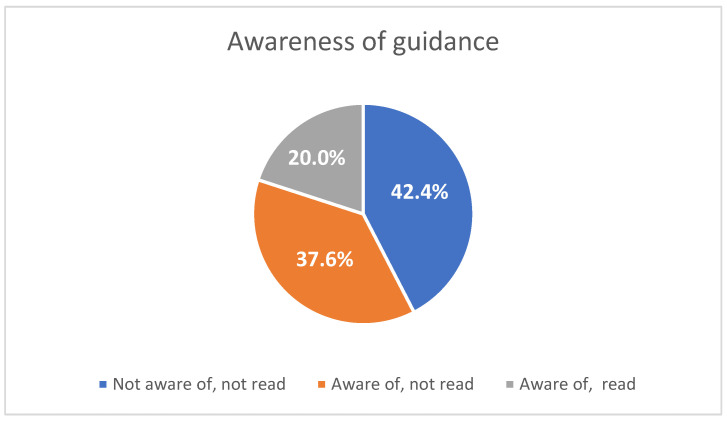
Participants’ awareness of the EASO-EFAD guidance on MNT for adults with obesity.

**Figure 2:**
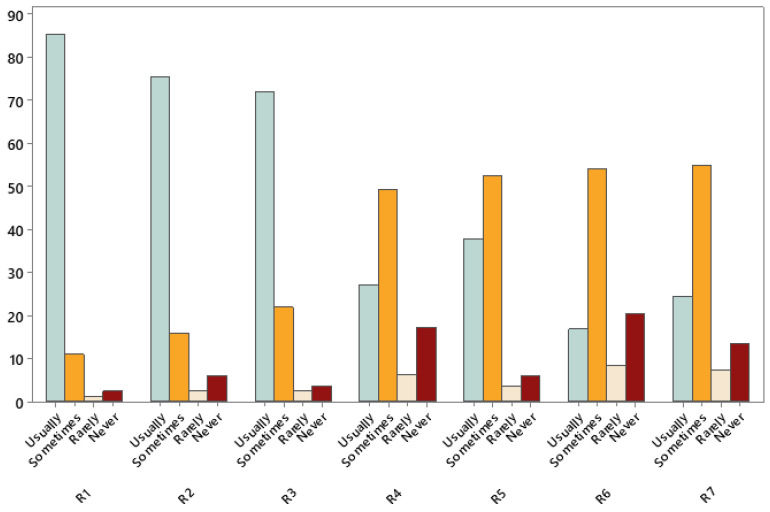
Extent to which recommendations are already in dietetic usual practice according to study participants. Data are expressed as percentages of responses. The following data are responses to the item, “In relation to your usual practice, please tick the box that best suits the frequency this is done in your practice”.

Dietitians’ and nutritionists’ responses to the questions of the survey are presented in Figure 1 and Figure 2 and Table 1. Specifically, over half (57.6%) were aware of the new EASO-EFAD guidelines, while 42.4% were not. However, only 20% of participants both were aware and had read the guidelines (Figure 1). Those who had read them were significantly more likely to possess post-graduate educational qualifications (*p* = 0.001) and have previous or current experience in running weight management programs (*p* = 0.004).

With regards to already existing national guidance, less than half of the participants (43.4%) stated that individualized MNT applied by registered dietitians (when these experts are available) to improve weight outcomes (see Table 1) in adults living with obesity is already part of national guidance. Similarly, only 40.0% and 48.2% reported that national guidance includes recommendations for intensive behavioral interventions targeting 7–15% or 5–7% weight loss for adults living with obesity and type 2 diabetes or impaired glucose tolerance (prediabetes) to improve specific outcomes, respectively (see Table 1). On the other hand, only a small percentage indicated that the national guidance they follow includes the portfolio dietary pattern as a means to improve established blood lipid targets; the DASH dietary pattern as a means to reduce body weight and waist circumference; partial meal replacements (replacing one to two meals/day is used as part of a calorie-restricted intervention) as a means to improve body weight, waist circumference, blood pressure, and glycemic control; and intermittent or continuous calorie restriction as a means to achieve similar short-term body weight reduction (16.9%, 38.5%, 21.0%, and 20.2%, respectively).

Interestingly, according to the responses of the participants, three recommendations (see Figure 2) were already more commonly used in clinical practice. These recommendations seemed to be integrated into the usual dietetic practices reported by the participants and included (a) individualized MNT by registered dietitians for adults living with obesity, (b) intensive behavioral interventions for adults living with obesity and type 2 diabetes, and (c) intensive behavioral interventions for adults living with obesity and impaired glucose tolerance (see Figure 2). The remaining recommendations were less commonly part of the usual dietetic practice (see Figure 2).

### 3.2. Interviews

The interviews identified a number of barriers to adopting the EASO-EFAD guidelines into clinical practice.

#### 3.2.1. Theme 1: Language Barriers Limit Accessibility

A recurring barrier was that the EASO-EFAD guidelines are currently available only in English, which restricts access for many European dietitians and nutritionists who are not fluent in English.

“The guidelines being only in English is a problem; it limits uptake from dietitians who don’t speak English fluently. If the guidelines were translated into other European languages, more dietitians could use them.”

#### 3.2.2. Theme 2: Rapidly Evolving Field Requires Updated and Flexible Recommendations

Dietitians emphasized that obesity management is a rapidly evolving field, and guidelines need to be updated regularly with wording that reflects current best practices.

“Obesity management is fast evolving, so recommendations and how they are worded need to keep pace.”

#### 3.2.3. Theme 3: Multidisciplinarity, Staffing, and Resource Constraints Limit Implementation

A major barrier is related to insufficient dietetic staff and resources to fully implement the guidelines. Dietitians stressed that managing obesity is complex and requires multidisciplinary teamwork and approach. However, lack of consistency and insufficient knowledge and training among other healthcare professionals, such as general practitioners (GPs), pose additional barriers.

“There is a lack of dietitians overall. Obesity is now recognized as a chronic disease by countries with subsidized healthcare, but the volume of patients living with obesity far exceeds available dietitian capacity.”

“There is a clear lack of knowledge and training in other professionals, which affects how they support dietary interventions. To effectively use some of the specific diets mentioned, reskilling of healthcare professionals is needed.”

#### 3.2.4. Theme 4: Emphasis on Health over Weight as an Outcome

Many dietitians, particularly from Ireland, reported that their focus is more on improving overall health rather than weight alone. This focus led to disagreement with some guideline wording, even though in practice, they were implementing individualized nutrition therapy.

“I focus more on health than weight as an outcome, so I could not agree with the wording of some of the statements, although in practice I am implementing individualized nutrition therapy.”

## 4. Discussion

The present study aimed to capture the perspectives of registered dietitians/nutritionists with experience on obesity management from four European countries (Greece, the Republic of Ireland, the UK, and the Netherlands) on the new European guidelines by EASO-EFAD for the dietetic management of obesity. The ultimate goal was to unravel potential needs or barriers toward the uptake and implementation of these guidelines into dietetic practice, in order to develop relevant strategies and close identified gaps.

The study showed that more than 40% of respondents were unaware of the guidelines, and only 20% both were aware of and had read the guidelines. These findings highlight the necessity of translating in local languages and intensively disseminating the guidelines to dietitians and nutritionists working in Europe, using diverse communication channels. Beyond the publication in the peer-reviewed literature [4], these should include scientific conferences and seminars for HCPs at local and international levels but also through patient group panels and audiences, using digital tools [16]. Previous studies have shown that dietitians favor digital education, although many of them rely on physical education, which indicates the need for promoting digital literacy in order to enhance the large participation of dietitians in digital training (e.g., seminars) [17,18]. Also, it has been previously highlighted that the incorporation of nutritional guidelines into the training (e.g., studies and practical placement) of clinicians could optimize clinical care [19,20,21]. As such, another important channel for the dissemination and actual uptake of the EASO-EFAD guidelines might be their use in the training process of HCPs such as dietitians, nutritionists, medical doctors, nurses, physical education instructors, and other healthcare professions.

Moreover, the decision-making bodies that develop national guidelines for the treatment of obesity and non-communicable diseases (e.g., the Ministry of Health) should be informed to adopt and adapt—if needed—the EASO-EFAD guidelines. Beyond that, the effectiveness and sustainability of the guidelines could be maximized through the development of a policy and food system that would enable the optimum implementation of the guidelines [22,23,24]. The European national health authorities and agencies should interact and align to adopt the guidelines and possibly develop electronic platforms to promote them [25,26]. In parallel, informing the food industry about the necessity of developing nutrient-rich, energy-low foods, in line with the guidelines, would provide a strong ground for the optimal uptake of the guidelines by the citizens [27].

Interestingly, although two of the EASO-EFAD recommendations are already used in dietetic/clinical practice in the participating countries, only a small percentage of the participants indicated that they are actually used in practice. More specifically, about one in five respondents replied that individualized medical nutrition therapy is used to improve anthropometric and health outcomes in adults living with obesity, whereas >20% reported that adult individuals with obesity and prediabetes receive intensive behavioral treatment to lose 5–7% of excessive weight. Additionally, although there is evidence to support that new nutritional strategies (e.g., portfolio diet, partial meal replacement, and intermittent fasting) may be beneficial for obesity and obesity-related health indices to certain adults living with obesity who did not comply with the traditional weight management techniques, only a small percentage of dietitians and nutritionists use them in practice. Previous reviews have suggested that selecting individualized approaches, after considering patients’ needs, risks, and preferences, may increase the effectiveness of dietetic intervention for weight loss and weight loss maintenance [28,29,30].

The study design was a strength, combining quantitative breadth with qualitative depth. The mixed-methods approach allowed us to capture both the prevalence of guideline awareness and the contextual barriers shaping implementation. Inclusion of participants from four European countries provided comparative insights across diverse healthcare contexts. However, this study has several limitations. First, recruitment was restricted to four European countries, limiting generalizability. National overweight and obesity guidelines differ substantially in content, translation, and availability; future studies should include broader representation. Second, recruitment through professional networks and snowball sampling may have favored professionals already engaged or fluent in English, introducing self-selection and language bias. Third, the qualitative component involved ten interviews. Although this number is consistent with published evidence indicating that thematic saturation can often be achieved with 6–12 interviews in thematic analysis, a larger sample could have provided even broader representation across settings [31,32]. No formal power calculation was performed, as the study was exploratory in nature. Finally, as responses were self-reported, participants’ perceptions of guideline content and personal practice may not fully reflect official national documents or observed behavior.

Future studies should recruit dietitians and nutritionists from a wider range of European countries, ensuring diversity in healthcare systems, languages, and practice settings. Partnerships with EFAD-affiliated national associations may mitigate self-selection bias. Expanding the qualitative component to 20–30 interviews would deepen representation across roles and settings. Verification of self-reported perceptions against national guideline documents, possibly via content analysis, would increase rigor. Implementation science frameworks could help address structural barriers, such as insufficient multidisciplinary infrastructure or inconsistent training among healthcare professionals. Practical dissemination strategies should include translation of the European Association for the Study of Obesity (EASO) and European Federation of the Associations of Dietitians (EFAD) guidelines into multiple languages, embedding them into dietetic and medical curricula and working with national authorities for local adaptation. Pilot initiatives, such as workshops in translated form or case-based multidisciplinary training, may provide evidence on feasibility and uptake.

## 5. Conclusions

In conclusion, this study provides novel insights into European dietitians’ and nutritionists’ awareness and perspectives on the new EASO-EFAD guidelines for obesity management. While certain recommendations align with current practice, overall awareness and uptake remain limited. Barriers include language accessibility, structural resource constraints, and differing views on outcome prioritization. Addressing these requires targeted dissemination, integration into training curricula, and alignment with national health systems. Future research should build on these exploratory findings with larger, representative samples and implementation-focused interventions. EFAD, with its expert group on obesity (ESND-Obesity), will develop strategies to promote the EASO-EFAD guidelines, aiming to improve weight management of adults living with obesity in Europe.

## Figures and Tables

**Table 1:** Extent to which recommendations are already in national guidance, according to study participants. Data are expressed as numbers (%). The following data are responses to the item, “In relation to your country’s guidance, tick the choice that best suits”.

In Relation to Your Country’s Guidance, Tick the Box That Best Suits	Already in Guidance	Not in Guidance	No Guidance
Recommendations [4]	N (%)		
R1. Adults living with obesity should receive individualized MNT by RDs to improve weight outcomes, glycemic control, blood lipids, BP	36 (43.4%)	21 (25.3%)	26 (31.3%)
R2. Adults living with obesity and type 2 diabetes should consider intensive behavioral interventions (7–15% weight loss)	32 (40.0%)	22 (27.5%)	26 (32.5%)
R3. Adults living with obesity and impaired glucose tolerance should consider intensive behavioral interventions (5–7% weight loss)	40 (48.2%)	18 (21.7%)	25 (30.1%)
R4. Portfolio dietary pattern to improve established blood lipid targets	12 (16.9%)	39 (54.94%)	20 (28.2%)
R5. Dietary Approaches to Stop Hypertension (DASH) dietary pattern to reduce body weight and waist circumference	30 (38.5%)	27 (34.6%)	21 (26.9%)
R6. Partial meal replacements to improve BW, WC, BP, glycemic control	17 (21.0%)	41 (50.6%)	23 (28.4%)
R7. Intermittent or continuous calorie restriction achieves similar short-term BW reduction	17 (20.2%)	40 (47.6%)	27 (32.1%)

MNT—medical nutrition therapy; RD—registered dietitian; BP—blood pressure; BW—body weight; WC—waist circumference.

## Data Availability

The data presented in this study are available on request from the corresponding author. The data are not publicly available due to ethical restrictions.

## Abbreviations

The following abbreviations are used in this manuscript:

EASO	European Association for the Study of Obesity
EFAD	European Federation of the Associations of Dietitians
MNT	Medical nutrition therapy
HCPs	Healthcare professionals

## Appendix A. Online Survey Questions

ESDN (Obesity) Survey for dietitians working in obesity management

This questionnaire relates to the recent paper by Hassapidou et al [4].

1. You will be shown a number of statements/recommendations from the paper, and asked your views about each.

Please note: even if you have not read the paper, your views are important. Thank you very much for taking part! We really appreciate it.

1. Which of the following best describes you in relation to this paper?
Not aware of it, have not read it
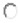
Aware of it, have not read it
Aware of it, have read it
2. In relation to national guidance or best practice for this statement, which of the following applies?
Adults living with obesity should receive individualized medical nutrition therapy provided by a registered dietitian (when available) to improve weight outcomes (body weight, BMI), waist circumference, glycemic control, established blood lipid targets, including LDL-C, triglycerides, and blood pressure.
This is already in my country’s national guidance or best practice for obesity
This is not in our current national guidance or best practice for obesity
We do not have national guidance or best practice for obesity
Other (please tell us more in Q23 below)
3. In relation to current practice for this statement, which of the following applies?
Adults living with obesity should receive individualized medical nutrition therapy provided by a registered dietitian (when available) to improve weight outcomes (body weight, BMI), waist circumference, glycemic control, established blood lipid targets, including LDL-C, triglycerides, and blood pressure.
This is usually done in practice
This is sometimes done in practice
This is rarely done in practice
This is never done in practice
4. In relation to national guidance or best practice for this statement, which of the following applies?
Adults living with obesity and type 2 diabetes should consider intensive behavioural interventions that target 7–15% weight loss to increase the remission of type 2 diabetes, reduce the incidence of nephropathy, obstructive sleep apnea and depression.
This is already in my country’s national guidance or best practice for obesity
This is not in our current national guidance or best practice for obesity
We do not have national guidance or best practice for obesity
Other (please tell us more in Q23 below)
5. In relation to current practice for this statement, which of the following applies?
Adults living with obesity and type 2 diabetes should consider intensive behavioural interventions that target 7–15% weight loss to increase the remission of type 2 diabetes, reduce the incidence of nephropathy, obstructive sleep apnea and depression.
This is usually done in practice
This is sometimes done in practice
This is rarely done in practice
This is never done in practice
6. In relation to national guidance or best practice for this statement, which of the following applies?
Adults living with obesity and impaired glucose tolerance (prediabetes) should consider intensive behavioral interventions that target 5–7% weight loss to improve glycemic control, blood pressure, blood lipids, reduce incident of type 2 diabetes, microvascular complications and cardiovascular and all-cause mortality.
This is already in my country’s national guidance or best practice for obesity
This is not in our current national guidance or best practice for obesity
We do not have national guidance or best practice for obesity
Other (please tell us more in Q23 below)
7. In relation to current practice for this statement, which of the following applies?
Adults living with obesity and impaired glucose tolerance (prediabetes) should consider intensive behavioral interventions that target 5–7% weight loss to improve glycemic control, blood pressure, blood lipids, reduce incident of type 2 diabetes, microvascular complications and cardiovascular and all-cause mortality.
This is usually done in practice
This is sometimes done in practice
This is rarely done in practice
This is never done in practice
8. In relation to national guidance or best practice for this statement, which of the following applies?
Portfolio dietary pattern to improve established blood lipid targets, including LDL-C, apo B, and non-HDL-C.
This is already in my country’s national guidance or best practice for obesity
This is not in our current national guidance or best practice for obesity
We do not have national guidance or best practice for obesity
Other (please tell us more in Q23 below)
9. In relation to current practice for this statement, which of the following applies?
Portfolio dietary pattern to improve established blood lipid targets, including LDL-C, apo B, and non-HDL-C.
This is usually done in practice
This is sometimes done in practice
This is rarely done in practice
This is never done in practice
10. In relation to national guidance or best practice for this statement, which of the following applies?
Dietary Approaches to Stop Hypertension (DASH) dietary pattern to reduce body weight and waist circumference.
This is already in my country’s national guidance or best practice for obesity
This is not in our current national guidance or best practice for obesity
We do not have national guidance or best practice for obesity
Other (please tell us more in Q23 below)
11. In relation to current practice for this statement, which of the following applies?
Dietary Approaches to Stop Hypertension (DASH) dietary pattern to reduce body weight and waist circumference.
This is usually done in practice
This is sometimes done in practice
This is rarely done in practice
This is never done in practice
12. In relation to national guidance or best practice for this statement, which of the following applies?
Partial meal replacements (replacing one to two meals/day as part of a calorie-restricted intervention) to reduce body weight, waist circumference, blood pressure and improve glycemic control.
This is already in my country’s national guidance or best practice for obesity
This is not in our current national guidance or best practice for obesity
We do not have national guidance or best practice for obesity
Other (please tell us more in Q23 below)
13. In relation to current practice for this statement, which of the following applies?
Partial meal replacements (replacing one to two meals/day as part of a calorie-restricted intervention) to reduce body weight, waist circumference, blood pressure and improve glycemic control.
This is usually done in practice
This is sometimes done in practice
This is rarely done in practice
This is never done in practice
14. In relation to national guidance or best practice for this statement, which of the following applies?
Intermittent or continuous calorie restriction achieved similar short-term body weight reduction.
This is already in my country’s national guidance or best practice for obesity
This is not in our current national guidance or best practice for obesity
We do not have national guidance or best practice for obesity
Other (please tell us more in Q23 below)
15. In relation to current practice for this statement, which of the following applies?
Intermittent or continuous calorie restriction achieved similar short-term body weight reduction.
This is usually done in practice
This is sometimes done in practice
This is rarely done in practice
This is never done in practice
16. If you answered ‘other’ to any of the questions above, please tell us more about your answers.

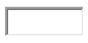

17. What would be difficult for you in changing your current advice to include the recommendations above?

18. What would help you overcome these difficulties?

## Appendix B. Topic Guide of Semi-Structured Interviews

Interview questions
1. Looking at the recommendations, what would be new to your practice? What would you have to do differently?
2. What, in your opinion, is the most important of the recommendations to implement?
3. Which of the recommendations would you say you (and colleagues) already include in their practice?
4. Thinking about making changes to your practice to implement the new recommendations, what would make change difficult for you?
5. What would help you overcome those difficulties?
6. What resources (e.g., podcasts, webinars, infographics), would help you in implementing the new recommendations?
7. Is there anything else you would like to add?
